# Natural compound Alternol exerts a broad anti-cancer spectrum and a superior therapeutic safety index *in vivo*

**DOI:** 10.3389/fphar.2024.1409506

**Published:** 2024-05-24

**Authors:** Chenchen He, Linlin Ma, Jeff Hirst, Fei Li, Hao Wu, Wang Liu, Jiang Zhao, Feng Xu, Andrew K. Godwin, Xiangwei Wang, Benyi Li

**Affiliations:** ^1^ Department of Radiation Oncology, The First Affiliated Hospital of Xi'An Jiaotong University, Xi'An, China; ^2^ Department of Urology, The University of Kansas Medical Center, Kansas City, KS, United States; ^3^ Department of Pathology and Laboratory Medicine, University of Kansas Medical Center, Kansas City, KS, United States; ^4^ Department of Physiology, Shenyang Pharmaceutical University, Shenyang, China; ^5^ Department of Urology, The Affiliated Hospital of Guangdong Medical University, Zhanjiang, China

**Keywords:** Alternol, anti-cancer drug, natural compound, prostate cancer, ovarian cancer

## Abstract

**Introduction:**

Alternol is a natural compound isolated from the fermentation of a mutated fungus. We have demonstrated its potent anti-cancer effect *via *the accumulation of radical oxygen species (ROS) in prostate cancer cells *in vitro* and *in vivo*. In this study, we tested its anti-cancer spectrum in multiple platforms.

**Methods:**

We first tested its anti-cancer spectrum using the National Cancer Institute-60 (NCI-60) screening, a protein quantitation-based assay. CellTiter-Glo screening was utilized for ovarian cancer cell lines. Cell cycle distribution was analyzed using flow cytometry. Xenograft models in nude mice were used to assess anti-cancer effect. Healthy mice were tested for the acuate systemic toxicity.

**Results:**

Our results showed that Alternol exerted a potent anti-cancer effect on 50 (83%) cancer cell lines with a GI_50_ less than 5 µM and induced a lethal response in 12 (24%) of those 50 responding cell lines at 10 µM concentration. Consistently, Alternol displayed a similar anti-cancer effect on 14 ovarian cancer cell lines in an ATP quantitation-based assay. Most interestingly, Alternol showed an excellent safety profile with a maximum tolerance dose (MTD) at 665 mg/kg bodyweight in mice. Its therapeutic index was calculated as 13.3 based on the effective tumor-suppressing doses from HeLa and PC-3 cell-derived xenograft models.

**Conclusion:**

Taken together, Alternol has a broad anti-cancer spectrum with a safe therapeutic index *in vivo*.

## Introduction

Ideally, any cancer therapy should be able to selectively kill cancer cells but spare normal/benign tissues. Unfortunately, current chemo-drugs like Taxanes are toxic to benign tissue/cells due to their non-selective acting mechanism (Hwang, 2012).

Alternol, also known as Alteronol, is a natural small molecule extracted from a mutant fungus, *Alternaria alternate* var. *monosporus*, obtained from Taxus brevifolia bark where the paclitaxel was originally isolated (Liu X. et al., 2007a). Preclinical testing in cell culture models and animal xenograft models showed that Alternol treatment caused cell cycle arrest and apoptotic cell death in multiple human and murine tumor cell lines or xenografts, potentially via a radical oxygen species (ROS)-dependent mechanism (Liu X. et al., 2007a; Liu Z. Z. et al., 2007b; Yao et al., 2012; Yeung et al., 2012; Liu et al., 2014; Zhu et al., 2014; Cong et al., 2015; Wang et al., 2016; Zuo et al., 2017; Ren et al., 2018; Ren et al., 2019; Bao et al., 2020; Ren et al., 2021). In our recent studies, we tested the anti-cancer effect of Alternol on multiple prostate cancer cell lines *in vitro* and their derived xenograft models in nude mice (Liu et al., 2020; Xu et al., 2020). We found that Alternol induced ROS-dependent cell death in most prostate cancer cell lines but spared the benign cells (Tang et al., 2014). Alternol-induced ROS accumulation was due to activation of xanthine oxidase in the cytosol (Xu et al., 2019). In addition, Alternol interacted with 14 cellular proteins including 6 ATP-producing mitochondrial proteins, resulting in ATP reduction *in vitro* and *in vivo* (Li et al., 2019). Most recently, we discovered that Alternol treatment elicited an immunogenic cell death that eliminates tumor growth of the untreated tumors by the host immune system (Li et al., 2021).

In this study, we expanded our Alternol testing to a broader cancer spectrum using the NCI-60 screening provided by the NCI Developmental Therapeutics Program (NCI-DTP). The screening results showed a strong growth inhibitory response in 50 (83.3%) cancer cell lines with a GI_50_ less than 5 μM. Meanwhile, 12 cancer cell lines displayed a lethal response to Alternol treatment at a concentration of less than 10 μM. These strong anti-cancer effects were also observed in 14 ovarian cancer cell lines. In an acute toxicity experiment in mice, a maximum tolerance dose was defined at about 665 mg/kg bodyweight. Therefore, the therapeutics index was calculated as 13.3 based on the effective dose at 10–50 mg/kg bodyweight in two types of human cancer xenograft models, HeLa, and PC-3. Our data suggest that Alternol is a potent anti-cancer agent with a broad cancer spectrum and a safe therapeutic index.

## Materials and methods

### Cell culture, chemical reagents, and antibodies

Human cervical cancer HeLa and prostate cancer PC-3 cell lines were purchased from ATCC and maintained in RPMI-1640, as described (Li et al., 2019). Ovarian clear cell carcinoma RMG-I cells were maintained in Ham’s F12 medium with 10% fetal bovine serum (FBS). A human high-grade serous ovarian cancer OVSAHO cell line was obtained from Millipore-Sigma and maintained in RPMI-1640 with 10% FBS. All 14 ovarian cancer cell lines used in the CellTiter-Glo screening were described in our previous publication (Hirst et al., 2018). Alternol compound with a 99.9% purity was a gift from Dr. Jiepeng Chen (Shantou Strand Biotech of China). N-acetylcysteine (N-Ac) was purchased from Sigma-Aldrich. Antibodies for PARP, caspase-3, and Actin were purchased from Cell Signal Technology. MitoSOX Green fluorescent dye (catalog #M36005) was purchased from ThermoFisher Scientific. The dihydroethidium (DHE)-based ROS detection assay kit (catalog #601290), propidium iodide (Catalog #14289), and penta-fluoro-benzene-sulfonyl fluorescein (PFBS, Catalog #10005983) were purchased from Cayman Chemicals.

### Flow cytometry and western blot assays

For cell cycle analysis, cells were seeded in a 6-well plate overnight, followed by treatment with the solvent or Alternol (10 μM final concentration). After trypsinization, cells in single-cell suspension in phosphate-buffered saline (PBS) were fixed with 70% cold ethanol. After three washes in PBS, cells were stained with propidium iodide (PI, 1 mg/mL)/0.1% Triton X-100 (v/v) staining solution containing DNase-free RNase A (0.2 mg/mL). Cell cycle distribution of PI-labeled cells was analyzed using a fluorescence-activated cell sorter (BD FACSAria IIIu), as described (Sun et al., 2007). The cell-cycle assays were performed three independent times with two technical replicates for each.

For fluorescent dye sorting with MitoSOX, DHE, and PFBS, cells were seeded in 12-well plates and treated with the solvent or Alternol (10 μM). At the end of treatment, cells were incubated with fluorescent dyes and harvested in single-cell suspension for flow cytometer analysis. The histograms for cell cycle distribution were generated with the FlowJ software.

For Western blot assays, cells seeded in 100-mm plates were treated as indicated in the figures. After treatment, cells were harvested into pellets and lysed in radioimmunoprecipitation assay (RIPA) buffer containing protease inhibitors (Pierce, Rockford, IL). An equal quantity of proteins from each sample was loaded onto the SDS-PAGE gels and transferred to Immuno-Blot™ PVDF membrane (Bio-Rad, Hercules, CA). After blocking in a Tris-buffered solution plus 0.1% Tween-20 (TBS-T) and 5% nonfat dry milk, membranes were incubated with primary antibodies overnight at 4C. Immunoreactive signals were developed in ECL solution (Santa Cruz Biotech).

### NCI-60 cell line cytotoxicity screening

Following drug addition, the plates are incubated for an additional 48 h at 37°C, 5% CO_2_, 95% air, and 100% relative humidity. For adherent cells, the assay is terminated by the addition of cold trichloroacetic acid (TCA). Cells are fixed *in situ* by the gentle addition of 50 µL of cold 50% (w/v) TCA (final concentration, 10% TCA) and incubated for 60 min at 4°C. The supernatant is discarded, and the plates are washed five times with tap water and air dried. Sulforhodamine B (SRB) solution (100 µL) at 0.4% (w/v) in 1% acetic acid is added to each well, and plates are incubated for 10 min at room temperature. After staining, unbound dye is removed by washing five times with 1% acetic acid and the plates are air dried. Bound stain is subsequently solubilized with 10 mM Trizma base, and the absorbance is read on an automated plate reader at a wavelength of 515 nm. For suspension cells, the methodology is the same except that the assay is terminated by fixing settled cells at the bottom of the wells by gently adding 50 µL of 80% TCA (final concentration, 16% TCA). Using the seven absorbance measurements: Tz = time zero, C = control growth, Ti = test growth in the presence of drug: [(Ti-Tz)/(C-Tz)] x 100 for concentrations for which Ti>/ = Tz growth. [(Ti-Tz)/Tz] x 100 for concentrations for which Ti < Tz cell death. GI_50_ = growth inhibition of 50% (GI_50_) is calculated from [(Ti-Tz)/(C-Tz)] x 100 = 50% reduction in the net protein increase in control cells during the drug incubation. TGI = total growth inhibition. LC_50_ = 50% reduction in the measured protein at the end of the drug treatment as compared to that at the beginning, a net loss of cells following treatment [(Ti-Tz)/Tz] x 100 = −50. Values are calculated for each of these three parameters if the level of activity is reached; however, if the effect is not reached or is exceeded, the value for that parameter is expressed as greater or less than the maximum or minimum concentration tested.

### CellTiter-Glo screening on ovarian cancer cells

Cells were seeded in 96-well plates overnight, followed by drug treatment at the concentrations indicated in the figures for 24 h. Cell viability was measured using CellTiter-Glo assay solution (Promega, Catalog #G9241) at 1:1 (v/v) to culture media for 1 h at 37C. Relative viability was established for the control (DMSO) and directly compared to the drug treatment, as described in our recent publication (Hirst et al., 2018).

### Acute systemic toxicity experiment in mice

An “up and down” approach was utilized to determine the minimum lethal and maximum nonlethal doses following the OECD guidelines (Buschmann, 2013). Healthy female mice at the age of 7 weeks were obtained from the vendor (Liaoning Changsheng Biotech) and housed in a 12/12 light circle with a regular diet. Alternol was dissolved in Linoleic acid for intraperitoneal injection at 100 μL in volume. The minimum lethal dose was defined as 1,275 mg/kg, and the maximum non-lethal dose was 665.6 mg/kg, and 3 doses were set between these two doses at an interval index of 0.85. The experimental mice were randomly divided into five groups, and then 1,083.8, 921.2, and 783.0 mg/kg dose groups were administered, and the reaction of the animals was observed for 3 days. At the end of the experiments, major organs were harvested for histological evaluation after H&E staining. The animal protocol was approved by the Institutional Animal Care and Use Committee (IACUC) at Shenyang Pharmaceutical University.

## Xenograft tumor models in nude mice

Athymic NCr-nu/nu male mice were purchased from Charles River Laboratory and were maintained following the Institutional Animal Care and Use Committee (IACUC) procedures and guidelines at Xi’An Jiaotong University. Xenograft tumors were established subcutaneously with human cervix cancer HeLa cells and prostate cancer PC-3 cells as described in our publication (Li et al., 2019). Briefly, 2.0 × 10^6^ exponentially grown cells in RPMI-1640 suspension were injected subcutaneously into the flanks of 6-week-old mice. Alternol was dissolved in a solvent that contained 20% DMSO in a PBS solution. When tumors were palpable (about 3.0 mm^3^), animals were treated via intraperitoneal injection of the solvent or Alternol every 2–3 days as indicated in the figures. Tumor growth was monitored by caliper measurement of the length (L) and the width (W). Tumor volumes were calculated as described previously (Tang et al., 2014).

## Data presentation and statistical methods

Quantitative data were presented as MEAN ± SEM (Standard error of the MEAN) from multiple repeated experiments. Data from Western blots, flow cytometry, and histological analysis were shown with representative images from 2-3 independent experiments. Statistical analyses were conducted using SPSS software with adequate approaches as described in the figure legends.

## Results

### Alternol exerts a strong and broad anti-cancer spectrum on multiple human cancer types

**FIGURE 1:**
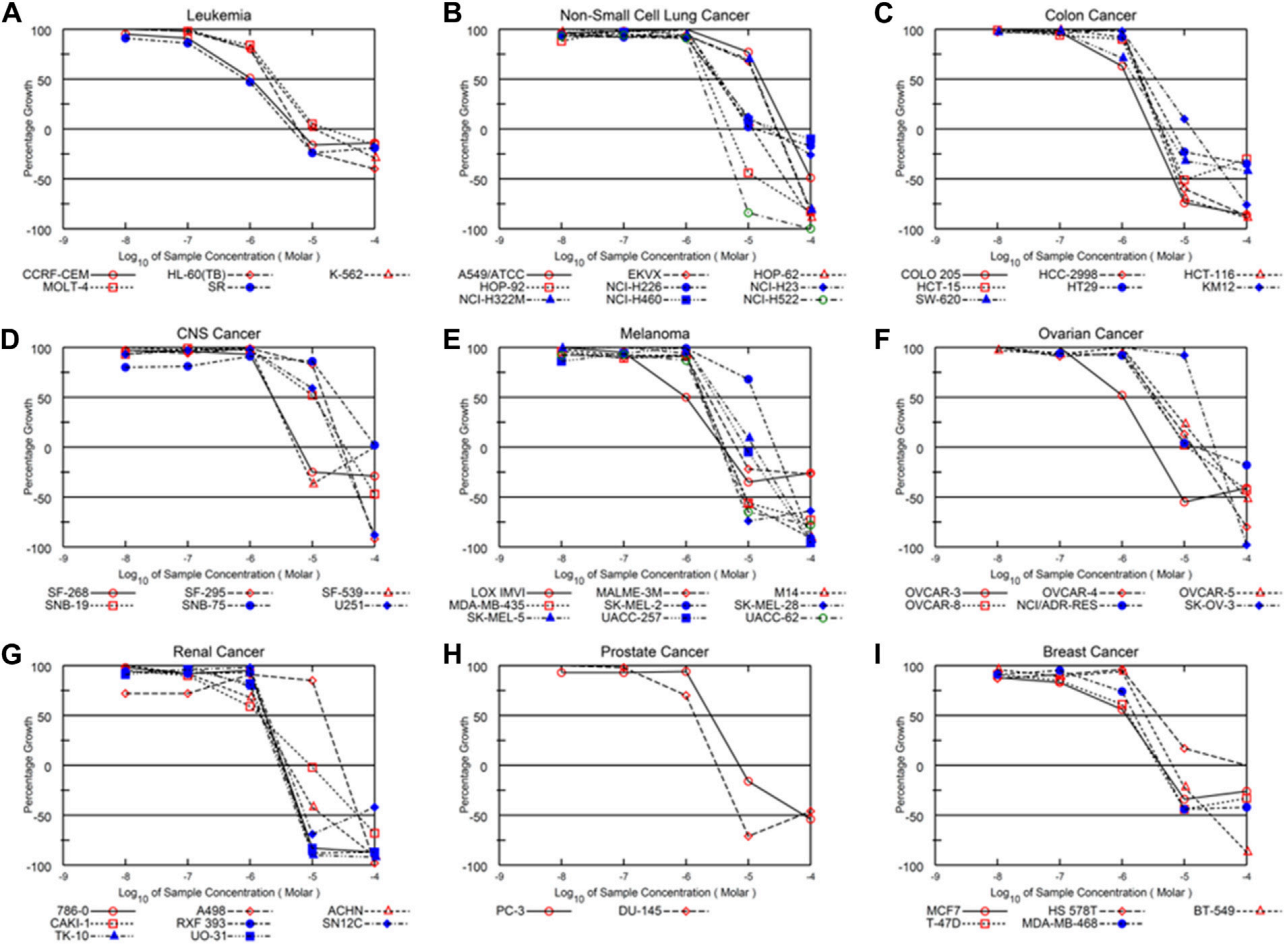
Alternol exerts a broader anti-cancer spectrum in NCI-60 screening. Graphic curves for the quantitative results of growth inhibitory effect on nine different cancer types **(A–I)** as indicated on the tope of each panel.

Alternol is a small natural compound purified from fermentation products of a mutant microorganism (Liu et al., 2020). We recently demonstrated its prostate cancer cell-preferential killing over benign prostate cells through a ROS-dependent mechanism (Tang et al., 2014; Xu et al., 2019), which was supported by the work of others (Yeung et al., 2012; Zuo et al., 2017). In collaboration with the NCI Developmental Therapeutic Program (NCI-DTP, NSC#D-783200, Experiment ID = 1503NS47), we conducted an NCI-60 screening with the sulforhodamine B (SRB)-based cell toxicity assay (Adan et al., 2016). The NCI-60 panel of human cancer cell lines was derived from nine common cancer types (Grever et al., 1992). We used five different drug concentrations (10 nM, 100 nM, 1 μM, 10 μM, 100 μM) for the toxicity experiments and calculated the growth inhibitory rate and cell death rate. Quantitative data of cell responses at each concentration were summarized in Supplementary Tables S1, S2, and the graphic data were shown in Figure 1. The GI_50_ and LC_50_ values for each cell line were summarized in Table 1. In general, the screening results showed that Alternol suppressed cell growth for all the NCI-60 panel lines with a GI_50_ (50% growth inhibition) at 0.835–26.7 μM and induced cell death in 31 (51.7%) cancer cell lines.

**TABLE 1:** Summary of NCI-60 cancer cell panel screening results.

Panel/Cell line	GI50	LC50
Leukemia		
CCRF-CEM	1.05E-06	> 1.00e-4
HL-60(TB)	1.93E-06	> 1.00e-4
K-562	2.36E-06	> 1.00e-4
MOLT-4	2.71E-06	> 1.00e-4
SR	3.50E-08	> 1.00e-4
Non-Small Cell Liung Cancer		
A549/ATCC	1.64E-05	> 1.00e-4
EKVX	1.31E-05	6.05E-05
HOP-62	3.10E-06	3.00E-05
HOP-92	2.38E-06	1.44E-05
NCI-H226	3.21E-06	> 1.00e-4
NCI-H23	3.31E-06	> 1.00e-4
NCI-H322M	1.36E-05	6.24E-05
NCI-H460	3.74E-06	> 1.00e-4
*NCI-H522	1.72E-06	6.43E-06
Colon Cancer		
*COLO 205	1.24E-06	6.69E-06
*HCC-2998	2.12E-06	8.70E-06
*HCT-116	1.97E-06	7.65E-06
HCT-15	1.93E-06	n/a
HT29	2.33E-06	> 1.00e-4
KM12	3.51E-06	5.00E-05
SW-620	1.60E-06	> 1.00e-4
CNS Cancer		
SF-268	2.32E-06	> 1.00e-4
SF-295	1.54E-05	5.74E-05
SF-539	2.42E-06	> 1.00e-4
SNB-19	1.06E-05	> 1.00e-4
SNB-75	2.67E-05	> 1.00e-4
U251	1.16E-05	5.51E-05
Prostate Cancer		
PC-3	2.52E-06	7.76E-05
DU-145	1.39E-06	n/a

Panel/Cell line	GI50	LC50
Melanoma		
LOX IMVI	9.76E-07	> 1.00e-4
MALME-3M	2.31E-06	> 1.00e-4
*M14	1.90E-06	8.80E-06
*MDA-MD-435	1.92E-06	9.17E-06
SK-MEL-2	1.30E-05	5.53E-05
*SK-MEL-28	1.83E-06	7.17E-06
SK-MEL-5	3.54E-06	3.78E-05
UACC-357	2.98E-06	3.14E-05
*UACC-62	1.75E-06	7.96E-06
Ovarian Cancer		
OVCAR-3	1.04E-06	n/a
OVCAR-4	3.51E-06	4.78E-05
OVCAR-5	4.55E-06	9.40E-05
OVCAR-8	3.36E-06	> 1.00e-4
NCI/ADR-RES	3.01E-06	> 1.00e-4
SK-OV-3	1.66E-05	5.59E-05
Renal Cancer		
*786-O	1.79E-06	6.55E-06
A498	1.55E-05	5.44E-05
ACHN	1.46E-06	1.46E-05
CAKI-1	1.41E-06	5.32E-05
*RXF 393	1.73E-06	6.17E-06
SN12C	1.93E-06	n/a
*TK-10	1.49E-06	5.79E-06
*UO-31	1.57E-06	6.33E-06
Breast Cancer		
MCF7	1.17E-06	> 1.00e-4
HS 578T	3.78E-06	> 1.00e-4
BT-549	2.38E-06	2.68E-05
T-47D	1.27E-06	> 1.00e-4
MDA-MB-468	1.61E-06	> 1.00e-4

50/60 (83.3%) GI50 < 4.55 mM; 12/60 (20%) LC50 < 10 mM.

**FIGURE 2:**
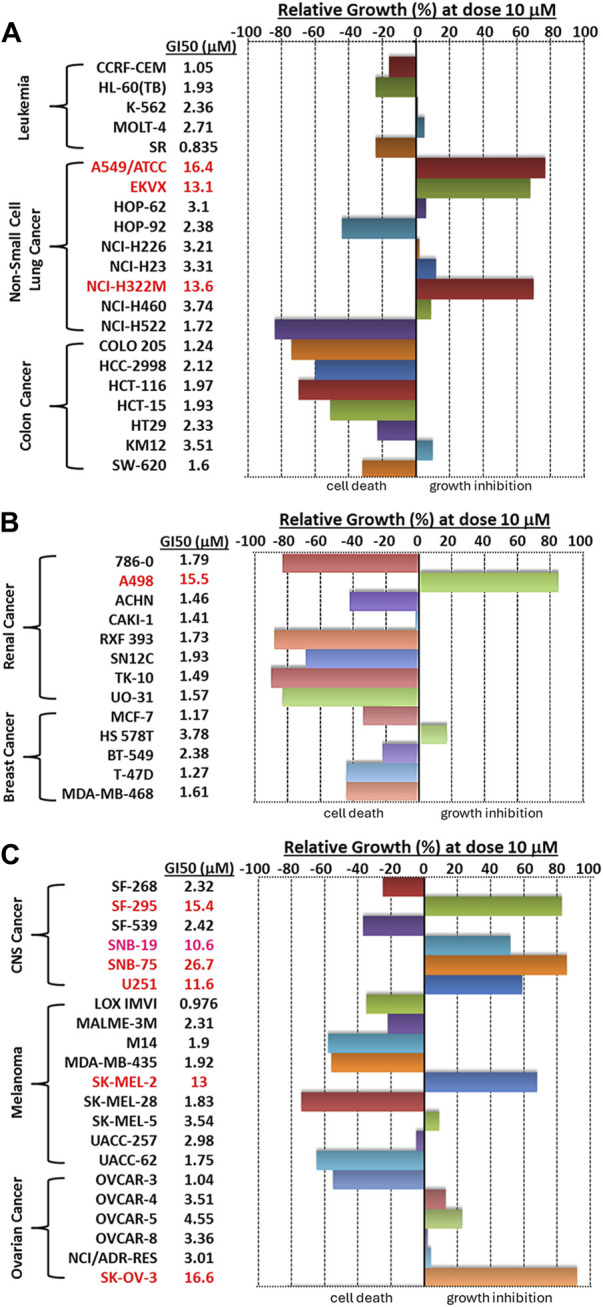
A bar graph summary of Alternol-induced growth inhibition on NCI-60 cell lines. The GI50 values on each cell line were listed on the left side of the graph.

We then visualized the growth inhibitory rates of each cell line at 10 μM Alternol treatment together with the GI_50_ value. As shown in Figure 2, all leukemia (5 lines), colon cancer (7 lines), and breast cancer (5 lines) cell lines showed a lethal response at 10 μM with a very low GI_50_ value (0.835–3.78 μM). Seven out of eight renal cancer cell lines, 8 out of nine melanoma cell lines, and five out of six ovarian cancer cell lines also showed a strong lethal response to Alternol treatment at 10 μM and their GI_50_ values were between 0.97–4.55 μM. However, renal cancer A498, melanoma SK-MEL-2, and ovarian cancer SK-OV-3 showed a slight inhibitory response with a GI_50_ value between 13–16.6 μM. In addition, six out of 9 NSCLC (HOP-62, HOP-92, H226, H23, H460, and H522) and two out of 6 CNS cancer (SF-268 and SF-539) cell lines showed a strong inhibitory response with a GI_50_ value between 2.32–3.74 μM. Of which HOP-92 and SF-539 cell lines also exerted a moderate lethal response (about 40% death rate). Conversely, three NSCLC lines (A549, EKVX, and H322M) and 4 CNS lines (SF-295, SNB-19, SNB-75, and U251) only showed a weak inhibitory response (<50%) with a GI_50_ value above 10 μM (10.6–26.7 μM). Therefore, 12 (20%) cancer cell lines showed a strong lethal response with an LC_50_ value between 1.72–9.4 μM, including NSCLC H522, colon cancer COLO205, HCC2998 and HCT116, renal cancer 786-O, RXF393, TK10, and UO31, melanoma SK-MEL-28 and UACC-62, as labeled in Table 1. In contrast, only 10 (16.7%) cancer cell lines (NSCLC A549, EKVX, NCI-H322M, renal cancer A498, CNS cancer SF-295, SNB-19, SNB-75, U251, Melanoma SK-MEL-2, and Ovarian cancer SK-OV-3) displayed a very weak inhibitory response with GI_50_ value between 11.6–26.7 μM. Taken together, 50 (83.3%) cancer lines showed a GI_50_ at ≤ 4.55 μM, of which 12 (24%) cancer lines showed a lethal response at an LC_50_ less than 10 μM. These data suggest that Alternol exerted a broad and potent anti-cancer spectrum.

### Alternol induces a distinct cell-specific response depending on cancer aggressiveness

**FIGURE 3:**
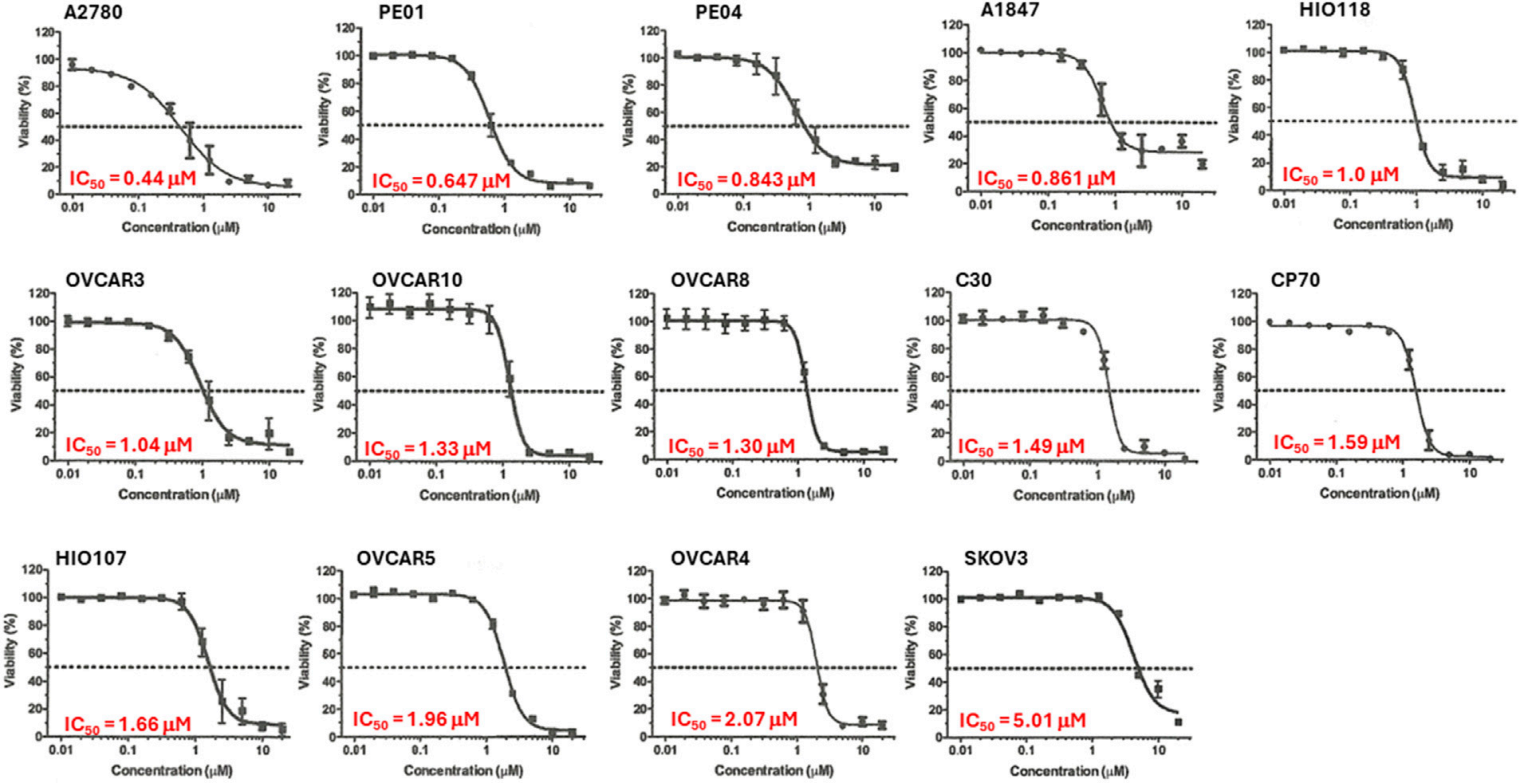
Alternol displayed a potent inhibitory effect on cell viability. Ovarian cancer cells as indicated were seeded in 96-well plates and subjected to CellTitder-Glo assay. The dotted line indicates the 50% inhibitory effect on cell viability.

We previously demonstrated that Alternol treatment reduced cellular ATP production by interrupting the mitochondrial TCA cycle (Li et al., 2019). Since ovarian cancer cell lines showed a diverse response pattern on the NCI-60 SRB screening assay (Figure 2), we then utilized a secondary assay, the ATP quantitation-based CellTiter-Glo assay (Ramos et al., 2023), to assess cell viability on 14 ovarian cancer cell lines. As shown in Figure 3, 13 (92.9%) out of 14 cell lines showed an IC_50_ between 0.44–2.07 μM with only 1 cell line SK-OV-3 with an IC_50_ at 5.01 μM.

**FIGURE 4:**
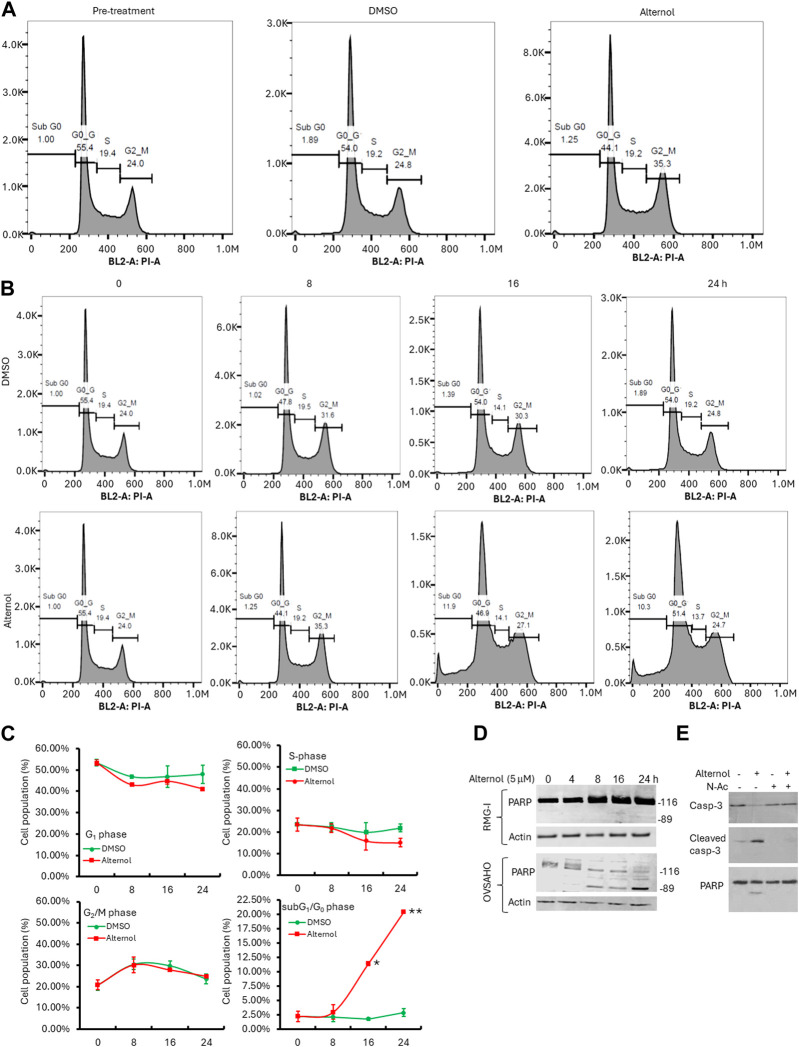
Alternol induces distinct responses depending on cancer cell aggressiveness. **(A,B)** Cell cycle analysis with flow cytometer in RMG-I (panel A) and OVSAHO (panel B) cells after Alternol treatment for up to 24 h. **(C)** Graphic summary of cell cycle distribution in OVSAHO cells after Alternol treatment. *p < 0.05; **p < 0.01, Student’s t-test. **(D)** Alternol induces PARP cleavage in OVSAHO but not RMG-I cells. Cells were treated with Alternol for up to 24 h and then harvested for anti-PARP immunoblotting assay. Actin blot was used as the protein loading control. **(E)** Alternol induces ROS-dependent Caspase-3 processing and PARP cleavage. OVSAHO cells were pre-treated with N-Ac (5 mM) for 30 min followed by Alternol treatment (5 μM) as indicated overnight. Cells were harvested for Western blot assay with antibodies to Caspase-3 and PARP. Actin blot was used as the protein loading control.

To understand if the cancer cell aggressiveness is related to the Alternol responsiveness, we selected the highly aggressive OVSAHO and the less aggressive RMG-I ovarian cancer cell lines, according to previous reports (Anglesio et al., 2013; Domcke et al., 2013). These 2 cell lines were treated with Alternol at different concentrations and periods. Our results showed that RMG-I cells exerted a cytostatic response without cell detachment or cell death after Alternol treatment at a concentration of up to 10 μM. In contrast, OVSAHO cells experienced a profound cell death after Alternol treatment at a concentration starting from 2.5 μM. Flow cytometry analysis revealed that RMG-I cells were arrested at the G_2_/M phase (Figure 4A) while OVSAHO cells had no obvious cell cycle disturbance but a significant accumulation of subG_1_/G_0_ cells (Figures 4B, C), a sign of apoptotic cell death (Ormerod et al., 1992). The apoptotic response in OVSAHO but not in RMG-I cells was confirmed using the gold standard marker, PARP cleavage (Figure 4D). Furthermore, the apoptotic response in OVSAHO cells was completely blocked by the pre-treatment of ROS scavenger N-Ac, as detected with Caspase-3 processing and PARP cleavage assays (Figure 4E), demonstrating a ROS-dependent apoptotic cell death in Alternol-treated aggressive cancer cells.

### Alternol induces mitochondrial ROS stress in aggressive cancer cells

**FIGURE 5:**
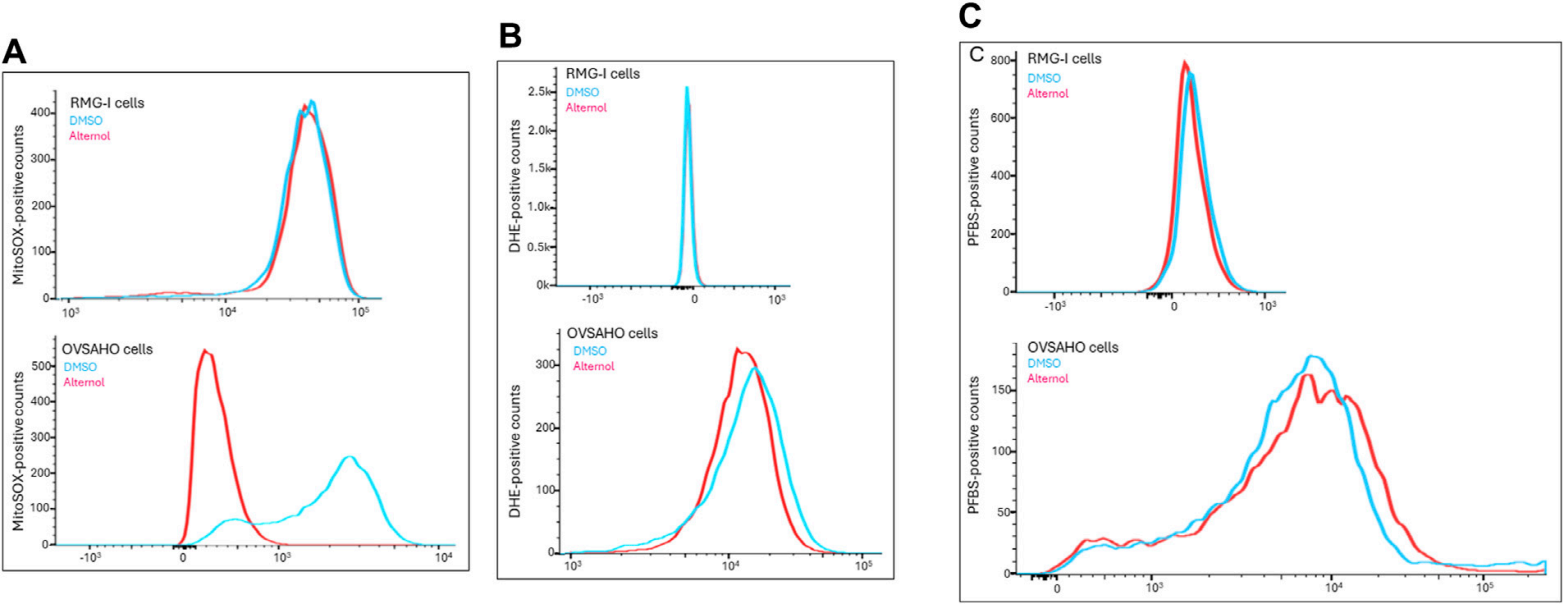
Detection of ROS probe distribution after Alternol treatment. RMG-I and OVSAHO cells were seeded in 6-well plates and treated with the solvent or Alternol (5 μM) for 4 h. After washing in PBS, cells were incubated with the fluorescent probes (5 μM), MitoSOX panel **(A)**, DHE panel **(B)**, or PFBS panel **(C)**, for 30 min followed by flow cytometer analysis of probe distribution.

Alternol was shown to induce cellular reactive oxygen species (ROS)-dependent apoptosis in multiple cancer cell lines (Liu et al., 2020). We sought to understand the ROS types induced by Alternol in OVSAHO cells with three different fluorescent probes, DHE for total superoxide (O_2_^−^) (Tarpey et al., 2004), MitoSOX for mitochondrial superoxide (Robinson et al., 2006), and PFBS for hydrogen peroxide (H_2_O_2_) (Maeda et al., 2004). As shown in Figure 5A, the Alternol treatment induced a remarkable curve shift of MitoSOX probe distribution in OVSAHO cells. Meanwhile, DHE but not PFBS probe distribution was slightly elevated after Alternol treatment in OVSAHO cells (Figures 5B, C). In contrast, RMG-I cells did not show any noticeable alteration of these probes (Figure 5). These data indicate that Alternol treatment mainly induced a mitochondrial superoxide-related ROS accumulation in the aggressive OVSAHO cells.

### Alternol possesses a safe therapeutic index *in vivo*

**FIGURE 6:**
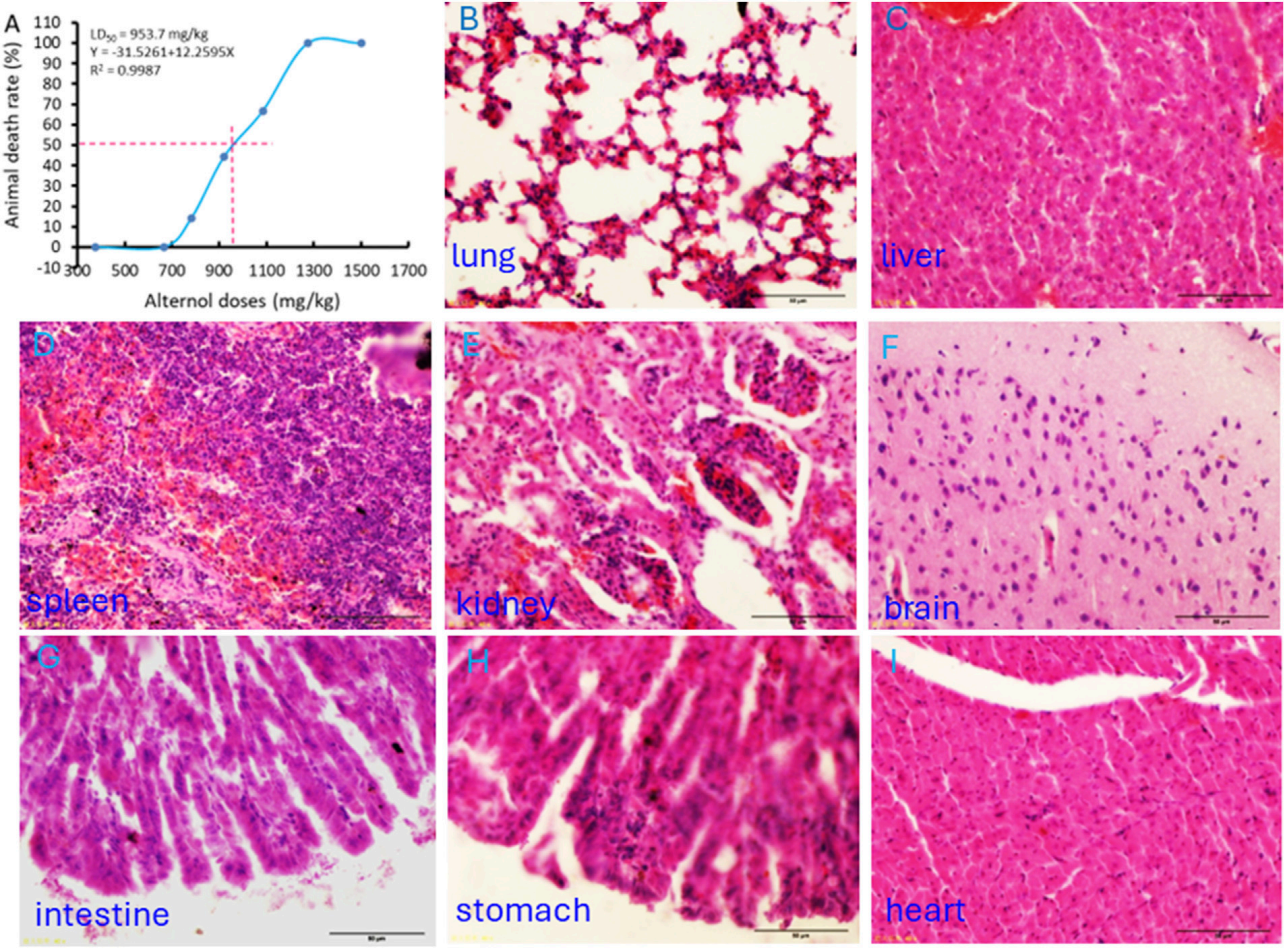
Acute systemic toxicity experiment in mice. **(A)** The “up and down” approach was utilized to determine the minimum lethal dose and maximum non-lethal dose. The LD_50_ value was calculated using the BLISS method. **(B–I)** Representative microscopic images of major organs as indicated harvested from the dead animal after the minimum lethal dosing.

The Alternol’s acute toxicity was tested in mice and the doses were selected using the up-and-down protocol (Dixon, 1965). The Bliss method was employed to calculate the value of LD_50_ (Rosiello et al., 1977). The summary of toxicity results is shown in Table 2 and the graphic image of the death rate in each dosing group is shown in Figure 6A. Briefly, there was no mortality at and below 665.6 mg/kg dosing group and mice began to die within 3 days after a single dose at 783 mg/kg. With the dose increased, the mortality increased. All mice died at the dosing group of 1,275 mg/kg with 20 min after injection. After Alternol injection at and above 783 mg/kg dosing, most animals immediately showed motionless, prone, drowsy, weak breathing, followed by respiratory depression and death. Few of them recovered within 3–5 days. In the groups received 665.6 mg/kg or less doses, there was no significant influence on body weight during the observation week after the single-dose treatment. The calculated LD_50_ for Alternol was 953.7 mg/kg for intraperitoneal administration with a 95% confidence interval of 764.81–1,189.3 mg/kg (Figure 4A). Also, the maximum tolerance dose (MTD) was defined as 665.6 mg/kg bodyweight.

**TABLE 2:** Summary of Alternol’s acute systemic toxicity in mice.

Dose (mg/kg)	log dose	Total animal (n)	Death animal	Death rate (%)	unit of probability
1,275	3.106	4	4	100	
1,083.8	3.017	3	2	66.6	5.43
921.2	2.964	9	4	44.4	4.859
783	2.894	7	1	14.3	3.931
665.6	2.823	6	0	0	

At the end of the experiments, major organs were harvested from the dead animals for histological evaluation. As shown in Figures 6B–F, interstitial hemorrhage was the major lesion observed in the lung, liver, kidney, and spleen. In the kidney, dilated renal tubules were observed but no abnormalities were noticed in the glomeruli. In addition, no histological damage was observed in the brain, heart, and intestine tissues (Figures 6G–I).

**FIGURE 7:**
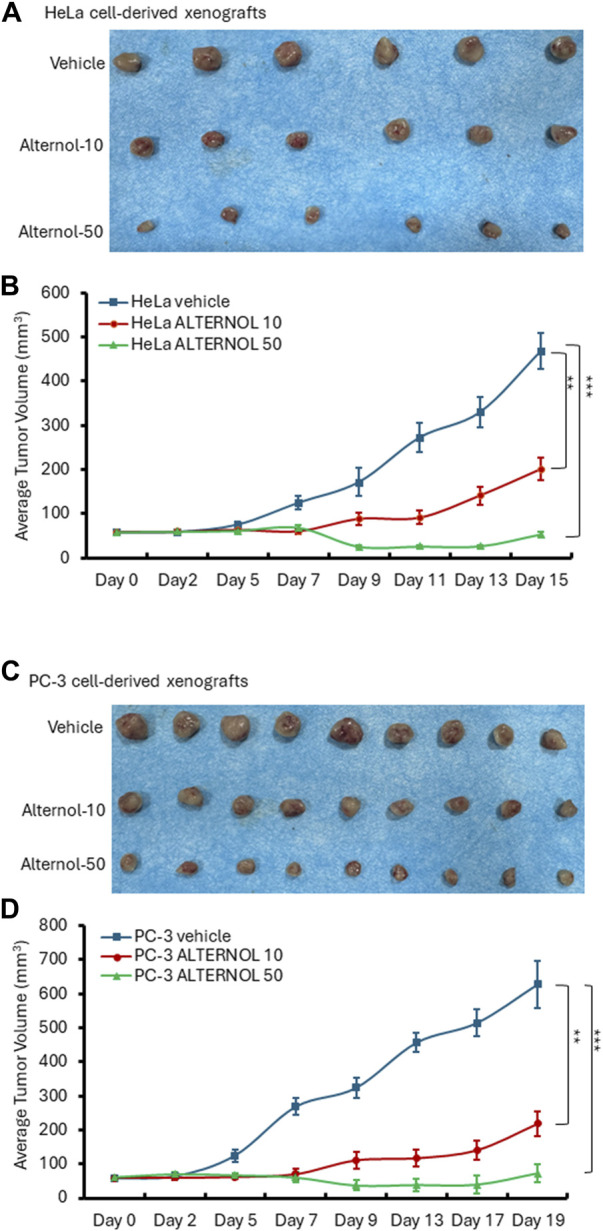
Alternol suppresses xenograft tumor growth in nude mice. HeLa cell-derived **(A,B)** or PC-3 cell-derived **(C,D)** xenografts were established in nude mice. Once the tumor was palpable, animals were treated with the solvent, or Alternol (10–50 mg/kg bodyweight) for 15–19 days as indicated. **p < 0.01; ***p < 0.001, ANOVA test.

We then tested Alternol’s anti-cancer effect on Hela cell-derived xenograft models in nude mice since it was reported that Alternol induced cell cycle arrest in HeLa cells (Yao et al., 2012). Once the xenograft tumors were palpable, animals were randomly assigned into three groups to receive treatment with the solvent or Alternol (10 or 50 mg/kg doses). As shown in Figures 7A, B, Alternol treatment at a dose of 50 mg/kg significantly suppressed Hela xenograft tumor growth compared to the vehicle control group, while Alternol at a dose of 10 mg/kg had only a slight reduction of tumor volume compared to the control group. Interestingly, PC-3 cell-derived xenograft models showed a very nice dose-dependent inhibitory effect on tumor growth at 10 and 50 mg/kg doses (Figures 7C, D). Therefore, the therapeutic index for Alternol was defined at 13 (MTD 665.6/50 effective dose), indicating a great potential for clinical development as a feasible anti-cancer therapy (Muller and Milton, 2012).

## Discussion

In this study, we provided strong and convincing data demonstrating Alternol’s broad spectrum of anti-cancer activities. With the help of the NCI-DTP program, we found that Alternol suppressed the cell growth in 50 (83.3%) cancer cell lines out of the NCI-60 panel with a GI_50_ of less than 4.55 μM. Among these 50 cancer cell lines, 12 cell lines showed a lethal response with an LC_50_ less than 10 μM. We also found that Alternol suppressed cellular ATP production in all 14 ovarian cancer cell lines tested in this study with an IC_50_ less than 5 μM. Further analysis revealed that Alternol induced a cytostatic effect in less aggressive cells but a lethal response in rapidly growing cells. These data indicate that rapid cell growth or proliferation is an important factor in cell death during Alternol treatment. In the acute toxicity experiment in mice, we defined the MTD dose at 665.6 mg/kg/bodyweight in mice, indicating a very low toxicity profile in animals. In considering the effective dose of <50 mg/kg depending on cancer cell lines, the therapeutic index of safety for *in vivo* treatment was calculated at 13.3, which represents a safe level for Alternol implication.

NCI-DTP screening project was established about 20 years ago to provide free-of-charge drug screening services (Grever et al., 1992). We chose the five-dose screen option with the SRB assay, which is a protein quantification method, to evaluate the cytotoxicity induced by the Alternol treatment. We also utilized the CellTiter-Glo assay, which measures cellular ATP level, for the screening of 14 ovarian cancer cell lines. The results from these two assays were very consistent for those overlapping ovarian cancer cell lines, indicating Alternol’s anti-cancer effect on cellular ATP production and viability.

Malignant tissues often harbor genetic alterations including mutation and copy number variation, responsible for their biological behaviors. Based on these genetic alterations and oncogene mutation status, ovarian cancer cell lines were recommended as either high-grade or unlikely high-grade types (Domcke et al., 2013). In this study, we tested 2 cell lines from these two distinct groups in responding to Alternol treatment. Our results showed a different behavior between high-grade OVSAHO and unlikely high-grade RMG-I cell lines. OVSAHO exhibited an apoptotic cell death response involving oxidative stress, but RMG-I only showed a cytostatic effect of G_2_/M cell cycle arrest. Rapid proliferating cancer cells like the high-grade OVSAHO line are most likely to suffer from oxidative stress (Deavall et al., 2012; Zhao et al., 2017) and, therefore, vulnerable to Alternol-induced ROS-dependent cell death, as described in our recent publication (Tang et al., 2014).

In conclusion, we characterized the natural compound Alternol as a broad anti-cancer agent against over 83% of human cancer cell lines based on the NCI-60 screening assay. We also demonstrated the Alternol compound has a safe therapeutic index of 13.3 with an MTD dose of over 660 mg/kg bodyweight. Alternol kills high-grade cancer cells by ROS-dependent mechanism but induces cytostatic effect on unlikely high-grade cancer cells. These data are useful for the potential clinical testing of the Alternol compound.

## Data Availability

The original contributions presented in the study are included in the article/Supplementary Material, further inquiries can be directed to the corresponding authors.
