# Supplementary figures and images for: Natural compound Alternol exerts a broad anti-cancer spectrum and a superior therapeutic safety index in vivo
Source: Front Pharmacol. 2024 May 24;15:1409506. doi: 10.3389/fphar.2024.1409506 (PMC11157072; doi:10.3389/fphar.2024.1409506)

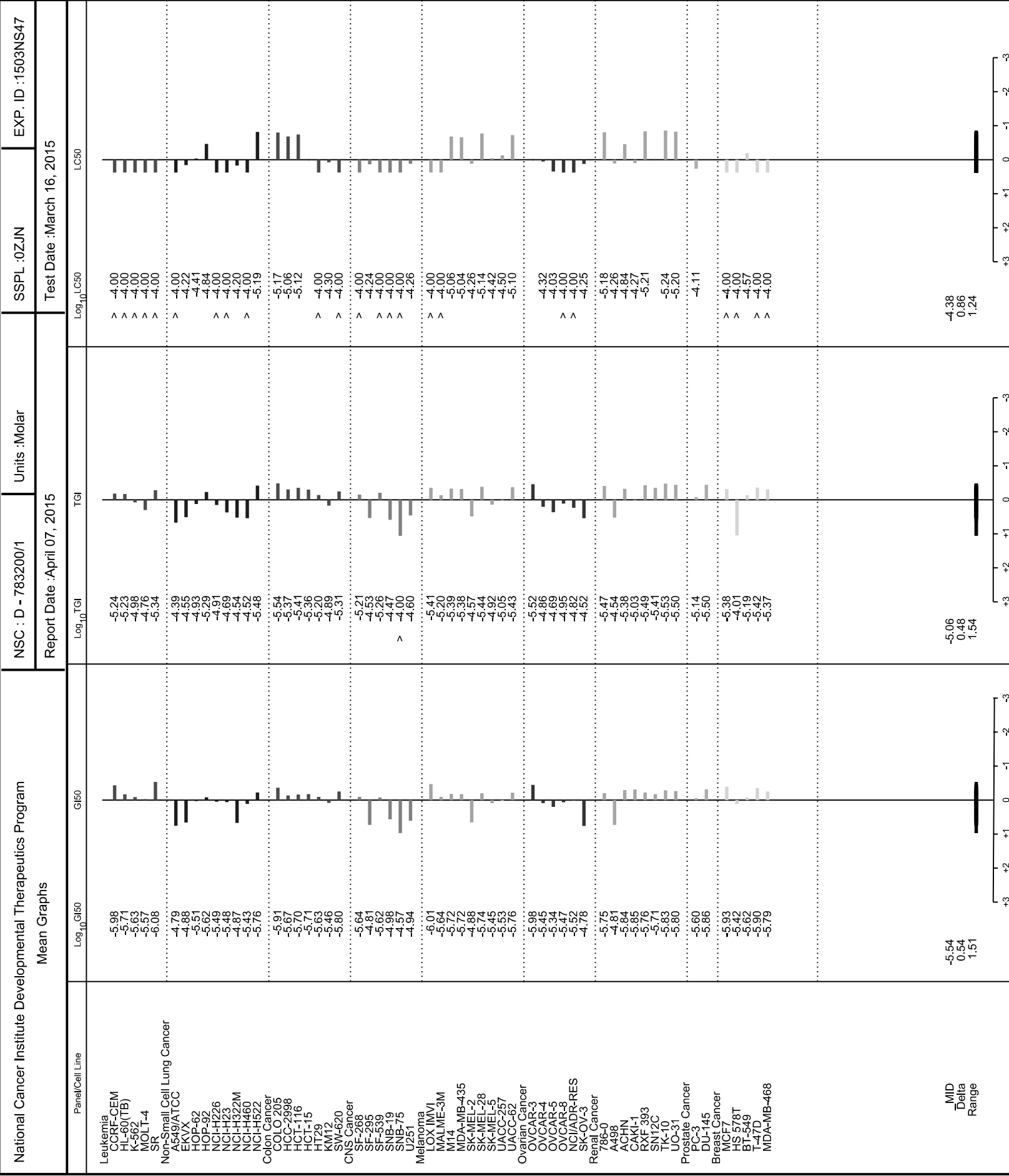

Supplement: Supplementary file 1 [file DataSheet2.PDF]
