# Supplementary material for: Natural compound Alternol exerts a broad anti-cancer spectrum and a superior therapeutic safety index in vivo
Source: Front Pharmacol. 2024 May 24;15:1409506. doi: 10.3389/fphar.2024.1409506 (PMC11157072; doi:10.3389/fphar.2024.1409506)
Supplement: Supplementary file 2 [file DataSheet1.PDF]

National Cancer Institute Developmental Therapeutics Program
In-Vitro Testing Results

NSC : D - 783200 / 1	Experiment ID : 1503NS47	Test Type : 08	Units : Molar
Report Date : April 07, 2015	Test Date : March 16, 2015	QNS :	MC :
COMI : Alternol	Stain Reagent : SRB Dual-Pass Related	SSPL : 0ZJN	

	Time		Log10 Concentration													
			Mean Optical Densities					Percent Growth								
Panel/Cell Line	Zero	Ctrl	-8.0	-7.0	-6.0	-5.0	-4.0	-8.0	-7.0	-6.0	-5.0	-4.0	GI50	TGI	LC50
**Leukemia**															
CCRF-CEM	0.718	3.005	2.886	2.802	1.895	0.602	0.618	95	91	51	-16	-14	1.05E-6	5.76E-6	> 1.00E-4
HL-60(TB)	1.168	3.389	3.420	3.341	2.936	0.888	0.702	101	98	80	-24	-40	1.93E-6	5.87E-6	> 1.00E-4
K-562	0.312	2.493	2.521	2.536	2.046	0.323	0.221	101	102	80	1	-29	2.36E-6	1.04E-5	> 1.00E-4
MOLT-4	0.920	3.287	3.290	3.242	2.916	1.037	0.776	100	98	84	5	-16	2.71E-6	1.74E-5	> 1.00E-4
SR	0.587	2.320	2.156	2.081	1.401	0.445	0.476	91	86	47	-24	-19	8.35E-7	4.57E-6	> 1.00E-4
**Non-Small Cell Lung Cancer**															
A549/ATCC	0.578	2.278	2.216	2.236	2.315	1.888	0.295	96	98	102	77	-49	1.64E-5	4.09E-5	> 1.00E-4
EKVX	0.773	1.692	1.651	1.620	1.640	1.397	0.132	96	92	94	68	-83	1.31E-5	2.82E-5	6.05E-5
HOP-62	0.705	1.899	1.858	1.838	1.806	0.780	0.078	97	95	92	6	-89	3.10E-6	1.16E-5	3.90E-5
HOP-92	1.510	1.979	1.924	1.977	2.011	0.849	0.263	88	100	107	-44	-83	2.38E-6	5.12E-6	1.44E-5
NCI-H226	0.883	2.181	2.104	2.079	2.175	0.906	0.734	94	92	100	2	-17	3.21E-6	1.24E-5	> 1.00E-4
NCI-H23	0.712	2.274	2.172	2.143	2.139	0.897	0.524	93	92	91	12	-26	3.31E-6	2.04E-5	> 1.00E-4
NCI-H322M	0.744	1.600	1.599	1.611	1.548	1.342	0.142	100	101	94	70	-81	1.36E-5	2.91E-5	6.24E-5
NCI-H460	0.194	2.306	2.336	2.247	2.417	0.382	0.176	101	97	105	9	-10	3.74E-6	3.03E-5	> 1.00E-4
NCI-H522	1.322	2.915	2.801	2.825	2.776	0.217	-0.002	93	94	91	-84	-100	1.72E-6	3.33E-6	6.43E-6
**Colon Cancer**															
COLO 205	0.610	1.961	1.987	1.923	1.460	0.160	0.083	102	97	63	-74	-86	1.24E-6	2.88E-6	6.69E-6
HCC-2998	0.598	2.592	2.528	2.522	2.656	0.240	0.081	97	96	103	-60	-87	2.12E-6	4.29E-6	8.70E-6
HCT-116	0.901	3.440	3.452	3.410	3.445	0.272	0.098	100	99	100	-70	-89	1.97E-6	3.88E-6	7.65E-6
HCT-15	0.340	2.334	2.316	2.213	2.140	0.167	0.237	99	94	90	-51	-30	1.93E-6	4.35E-6	.
HT29	0.278	1.810	1.865	1.857	1.693	0.214	0.182	104	103	92	-23	-35	2.33E-6	6.30E-6	> 1.00E-4
KM12	0.601	3.032	3.052	3.028	2.994	0.833	0.147	101	100	98	10	-76	3.51E-6	1.29E-5	5.00E-5
SW-620	0.330	2.316	2.257	2.276	1.739	0.226	0.190	97	98	71	-32	-42	1.60E-6	4.92E-6	> 1.00E-4
**CNS Cancer**															
SF-268	0.581	2.054	2.009	2.000	1.950	0.438	0.410	97	96	93	-25	-29	2.32E-6	6.17E-6	> 1.00E-4
SF-295	1.053	2.999	2.949	2.886	2.982	2.664	0.083	97	94	99	83	-92	1.54E-5	2.97E-5	5.74E-5
SF-539	0.915	2.602	2.622	2.543	2.672	0.575	0.914	101	97	104	-37	.	2.42E-6	5.46E-6	> 1.00E-4
SNB-19	0.798	2.209	2.115	2.192	2.167	1.537	0.423	93	99	97	52	-47	1.06E-5	3.36E-5	> 1.00E-4
SNB-75	1.427	2.705	2.454	2.464	2.589	2.520	1.458	80	81	91	86	2	2.67E-5	> 1.00E-4	> 1.00E-4
U251	0.727	2.383	2.270	2.339	2.346	1.709	0.086	93	97	98	59	-88	1.16E-5	2.52E-5	5.51E-5
**Melanoma**															
LOX IMVI	0.258	1.608	1.638	1.536	0.927	0.168	0.192	102	95	50	-35	-26	9.76E-7	3.86E-6	> 1.00E-4
MALME-3M	0.878	1.341	1.307	1.294	1.301	0.683	0.642	93	90	91	-22	-27	2.31E-6	6.37E-6	> 1.00E-4
M14	0.463	1.835	1.801	1.720	1.721	0.193	0.040	98	92	92	-58	-91	1.90E-6	4.09E-6	8.80E-6
MDA-MB-435	0.407	2.412	2.332	2.184	2.249	0.181	0.109	96	89	92	-56	-73	1.92E-6	4.20E-6	9.17E-6
SK-MEL-2	1.533	2.737	2.745	2.776	2.731	2.356	0.139	101	103	99	68	-91	1.30E-5	2.69E-5	5.53E-5
SK-MEL-28	0.451	1.377	1.413	1.418	1.323	0.116	0.163	104	104	94	-74	-64	1.83E-6	3.62E-6	7.17E-6
SK-MEL-5	0.791	3.007	2.990	3.037	3.017	0.980	0.058	99	101	100	9	-93	3.54E-6	1.21E-5	3.78E-5
UACC-257	1.543	2.347	2.235	2.297	2.344	1.470	0.065	86	94	100	-5	-96	2.98E-6	9.00E-6	3.14E-5
UACC-62	0.749	2.226	2.101	2.121	2.032	0.262	0.168	92	93	87	-65	-78	1.75E-6	3.73E-6	7.96E-6
**Ovarian Cancer**															
OVCAR-3	0.452	1.700	1.748	1.748	1.096	0.203	0.265	104	104	52	-55	-41	1.04E-6	3.04E-6	.
OVCAR-4	0.794	1.959	1.987	1.851	1.887	0.951	0.159	102	91	94	13	-80	3.51E-6	1.39E-5	4.78E-5
OVCAR-5	0.697	1.549	1.521	1.489	1.566	0.893	0.335	97	93	102	23	-52	4.55E-6	2.03E-5	9.40E-5
OVCAR-8	0.711	2.499	2.494	2.503	2.552	0.751	0.408	100	100	103	2	-43	3.36E-6	1.12E-5	> 1.00E-4
NCI/ADR-RES	0.629	2.252	2.250	2.161	2.128	0.694	0.514	100	94	92	4	-18	3.01E-6	1.51E-5	> 1.00E-4
SK-OV-3	0.869	1.656	1.656	1.652	1.680	1.589	0.019	100	100	103	92	-98	1.66E-5	3.04E-5	5.59E-5
**Renal Cancer**															
786-0	0.702	2.417	2.380	2.286	2.331	0.122	0.092	98	92	95	-83	-87	1.79E-6	3.43E-6	6.55E-6
A498	1.419	2.043	1.868	1.868	1.985	1.947	0.022	72	72	91	85	-98	1.55E-5	2.90E-5	5.44E-5
ACHN	0.728	2.745	2.757	2.590	2.102	0.423	0.067	101	92	68	-42	-91	1.46E-6	4.16E-6	1.46E-5
CAKI-1	0.629	3.090	2.960	2.842	2.086	0.618	0.200	95	90	59	-2	-68	1.41E-6	9.33E-6	5.32E-5
RXF 393	0.582	1.199	1.165	1.147	1.156	0.071	0.078	94	92	93	-88	-87	1.73E-6	3.27E-6	6.17E-6
SN12C	0.641	2.248	2.133	2.182	2.209	0.199	0.371	93	96	98	-69	-42	1.93E-6	3.85E-6	.
TK-10	0.852	2.082	2.095	2.081	1.825	0.084	0.067	101	100	79	-90	-92	1.49E-6	2.93E-6	5.79E-6
UO-31	0.973	2.591	2.439	2.526	2.308	0.168	0.125	91	96	82	-83	-87	1.57E-6	3.16E-6	6.33E-6
**Prostate Cancer**															
PC-3	0.693	1.989	1.899	1.898	1.911	0.586	0.317	93	93	94	-16	-54	2.52E-6	7.22E-6	7.76E-5
DU-145	0.374	1.753	1.778	1.721	1.343	0.109	0.204	102	98	70	-71	-46	1.39E-6	3.15E-6	.
**Breast Cancer**															
MCF7	0.383	2.163	1.950	1.856	1.382	0.253	0.282	88	83	56	-34	-26	1.17E-6	4.20E-6	> 1.00E-4
HS 578T	0.945	2.027	1.883	1.935	1.979	1.126	0.943	87	91	96	17	.	3.78E-6	9.72E-5	> 1.00E-4
BT-549	1.084	2.533	2.475	2.372	2.441	0.842	0.143	96	89	94	-22	-87	2.38E-6	6.41E-6	2.68E-5
T-47D	0.882	1.909	1.812	1.758	1.509	0.494	0.593	91	85	61	-44	-33	1.27E-6	3.81E-6	> 1.00E-4
MDA-MB-468	0.913	1.818	1.741	1.769	1.585	0.515	0.532	91	95	74	-44	-42	1.61E-6	4.27E-6	> 1.00E-4
